# Prior knowledge in recalling arguments in bioethical dilemmas

**DOI:** 10.3389/fpsyg.2015.01292

**Published:** 2015-09-08

**Authors:** Hiemke K. Schmidt, Martin Rothgangel, Dietmar Grube

**Affiliations:** ^1^Educational Psychology, Department of Education, Faculty I: School of Educational and Social Sciences, Carl von Ossietzky University of OldenburgGermany; ^2^Department of Religious Education, Faculty of Protestant Theology, University of ViennaVienna, Austria

**Keywords:** prior knowledge, multiple domains, interest, recall, bioethical dilemma

## Abstract

Prior knowledge is known to facilitate learning new information. Normally in studies confirming this outcome the relationship between prior knowledge and the topic to be learned is obvious: the information to be acquired is part of the domain or topic to which the prior knowledge belongs. This raises the question as to whether prior knowledge of various domains facilitates recalling information. In this study 79 eleventh-grade students completed a questionnaire on their prior knowledge of seven different domains related to the bioethical dilemma of prenatal diagnostics. The students read a text containing arguments for and arguments against prenatal diagnostics. After 1 week and again 12 weeks later they were asked to write down all the arguments they remembered. Prior knowledge helped them recall the arguments 1 week (*r* = 0.350) and 12 weeks (*r* = 0.316) later. Prior knowledge of three of the seven domains significantly helped them recall the arguments 1 week later (correlations between *r* = 0.194 and 0.394). Partial correlations with interest as a control item revealed that interest did not explain the relationship between prior knowledge and recall. Prior knowledge of different domains jointly supports the recall of arguments related to bioethical topics.

## Introduction

Prior knowledge is defined as all the knowledge one has before learning about a particular topic. As Dochy et al. (1999) point out, it facilitates learning new information. They estimate between 30 and 60% of the variance in learning outcomes is explained by prior knowledge. At first this seems plain and simple; however, after taking a closer look at definitions of prior knowledge and research on prior knowledge the question arises as to whether this definition includes broader concepts or whether prior knowledge is helpful with well-delimited content and domains only.

Dochy (1992) considers broad concepts such as “world knowledge” and “background knowledge” as prior knowledge. In his research he distinguishes between domain-specific knowledge and subject-oriented knowledge. Alexander et al. (1994a) use this classification as well but refer to subject-oriented knowledge as topic-knowledge. Both consider domain-specific knowledge as knowledge of a broader subject area, whereas topic knowledge or subject-oriented knowledge is knowledge of a specific topic within a subject. Dochys examples of domains include psychology, economics and law, which can be divided into subdomains, for instance, economics can be divided into accounting and finance. This is where the distinction of topic knowledge becomes difficult. A topic in the domain of law might be constitutional law in general or one legal paragraph in particular.

Research on prior knowledge often is on mathematical or scientific topics (Alexander and Murphy, 1998; DeMarie et al., 2004). The advantage of investigating prior knowledge of such topics is that they are well-structured and clearly defined in terms of related topics. It is easy to allocate to these topics the domain-specific prior knowledge necessary to acquiring subsequent knowledge. Extensive research has confirmed the great importance of acquiring domain-specific and topic knowledge; however, topics requiring clearly defined prior knowledge may cause problems in some school subjects and for some topics.

While most research on the relevance of prior knowledge for the acquisition of subsequent knowledge has been conducted for mathematical and scientific topics, little is known about the effectiveness of prior knowledge in social sciences and linguistics. Questions arise as to what would be considered necessary as well as adequate prior content knowledge to understanding more advanced concepts in these subjects. Dochy (1992) claims that, broad concepts stand in contrast to the relatively strict distinction between topic knowledge and domain knowledge. The contrast between these is illustrated when considering the various operationalizations of prior knowledge employed in different studies. In some studies, for example, prior knowledge is mere familiarity with a concept; in others it is the duration of instruction on a topic. In some studies participants assess their prior knowledge themselves; in others prior knowledge is assessed by researchers through questionnaires covering the understanding of broad and/or specific topics as well as the learning processes involved (cf. Kaakinen et al., 2003; DeMarie et al., 2004; Levin and Arnold, 2004; Clarke et al., 2005; Kobayashi, 2009; Miller and Keenan, 2009; Rittle-Johnson et al., 2009; Cromley et al., 2010; Schneider et al., 2011; Toth et al., 2011). Here, one can see that the definition of prior knowledge is not very clear. The questions to be addressed in this study are as follows.

Can prior content knowledge be clearly allocated per topic as seems the case in mathematical and scientific topics? Does prior knowledge facilitate learning concepts in social sciences? Is the distinction between domain-specific and topic knowledge helpful with subjects from social or linguistic sciences? Is a broader concept of prior knowledge needed for these subjects?

Topics related to social sciences can be difficult to allocate to one domain. Consequently, they are problematic when one tries adapting the categorization of domain-specific and topic knowledge. For example, the invention of the loom is a topic of history education and is allocated to the domain “history” and the subdomain “industrialization.” To understand concepts revolving around the loom and its importance in society, prior knowledge of technical aspects of weaving (e.g., needlework) and of economics might be helpful, too. This assumed addition of prior knowledge from different domains can be assumed for many less structured topics. Another example of a topic which is difficult to categorize but is taught in biology and religion education is the bioethical dilemma of prenatal diagnostics (PND). PND is a common topic of public discussion. It seems to be related to the domain of medicine; therefore it sometimes is addressed in biology class. To understand the biological part of PND prior knowledge of biology and medicine such as pregnancy and embryonic development is relevant. However, public discussion on PND tends to focus on the conflict between women's right to self-determination and the fetus's right to life. These are topics from the domain of law. To understand why this conflict launches animated discussion, knowledge is needed of various concepts of the human being, that is, theories about what a human essentially is and when human life begins. These theories belong to the domains of philosophy and religion. Such a topic seems to involve numerous domains; therefore it is more complex and less structured than a topic involving only one domain and it requires prior knowledge from those domains to be understood.

In research there are definitions on when a topic or problem is ill-defined or ill-structured. Spiro et al. (1992) describe an ill-structured knowledge domain with two characteristics. First, each case of knowledge application involves the interaction and involvement of multiple, sometimes complex conceptual structures which might be widely different from each other. Second, every case of application varies in what kind of knowledge and domains might need to be involved, even if the different cases seem to be similar. This definition fits bioethical dilemmas like PND, since such a topic involves multiple knowledge domains or concepts and each case of discussion whether to use PND varies in which kind of knowledge fits the concerns and considerations the parents or discussants have. A related definition to the one above is the one of ill-structured problems. Ill-structured problems are characterized by the fact that there are multiple solutions. The criteria to appraise these solutions are often unclear or not easy to determine. Kitchener (1983) uses this definition on everyday life and social problems.

The question is whether prior knowledge is helpful in ill-structured domains. Ohst et al. (2014) showed that unsystematic prior knowledge needs to be systematized before the actual learning process can successfully take place. Since prior knowledge on ill-structured, complex topics might be unsystematic and—especially in public discussions on bioethical dilemmas—intuitive, prior knowledge might not be as helpful for learning with complex topics as it is in topics from only one domain. On the other hand Heit et al. (2004) showed that new knowledge not congruent with a category is even learned easier because it attracts attention in the learner. Since school subjects are often regarded as a domain, an ill-structured, complex topic relating to different domains might attract a lot of attention.

Research on the prior knowledge of topics from the domains of language education and social sciences is scarce. These topics seem to be complex in terms of determining the appropriate amount and content of domain-specific prior knowledge. Ostensibly, there is no research on topics requiring prior knowledge from more than one domain. In this study, we investigate the role of content prior knowledge of a variety of domains in a learning situation on the complex topic of PND. Various domains of content prior knowledge which might be helpful for learning are considered. First, students' prior knowledge of these different domains is tested. Next, students read a text with arguments for and against PND and later are asked to recall the arguments presented in this text. The first question to be addressed is whether all prior knowledge from the different domains together facilitates recall. The second question is whether prior knowledge of these individual domains facilitates recall.

Additional variables that influence learning are assessed in this study as well. Interest is one of them. Interest in a topic or domain facilitates learning, and often is closely related to prior knowledge (cf. Alexander et al., 1994b, 1995; Tobias, 1994; Naceur, 2001; Ainley et al., 2002; Falk and Adelman, 2003); however, interest on its own can have a profound impact on learning outcomes. Especially when learning about complex topics, interest may carry more weight than past research has shown it to have on simpler topics. Does interest in a topic explain learning outcomes better than prior knowledge?

Although Dochy (1992) lists a variety of sometimes very broad concepts of prior knowledge, the difference between domain-specific prior knowledge and common knowledge might be small. Common knowledge is knowledge often learned during formal education. It is the sum of all knowledge someone might have, therefore broad, from multiple domains and often shared by other people. In this study we use a test on crystallized intelligence (Liepmann et al., 2007), since this construct comes close to the idea of common knowledge. Since common knowledge comprises multiple domains, and our expectations are that with ill-defined, complex topics content prior knowledge comes from a multitude of different domains, maybe in this case common knowledge and prior knowledge are not different at all. The question arises as to whether common knowledge is a better predictor than prior knowledge stemming from a variety of domains. Perhaps with complex topics common knowledge—not prior knowledge—explains recall. Finally, reading ability may be important for learning various topics, as knowledge often is acquired by reading texts. Does reading ability influence outcomes of learning complex topics more than domain-specific prior knowledge?

Complex topics are taught to students to help them relate school-based learning to real life problems. Learners might need such information in their future lives (Scherb, 2005; Kultusministerkonferenz, 2006; Reitschert, 2007). Ideally knowledge acquired in the classroom is remembered long after leaving school. Some studies have investigated whether long-term recall is facilitated by prior knowledge (cf. Hall and Edmondson, 1992; Gilabert et al., 2005); however, follow-up assessment never was conducted after more than 1 week. This interval of 1 week does not agree with school life intervals, during which knowledge needs to be remembered. Another question in this study is if long-term recall of knowledge also is facilitated by prior knowledge.

The research questions of this study are summarized as follows. First, the role of domain-specific prior knowledge in recollecting information on a complex topic is in question.

1. Do prior knowledge in general and recall correlate?
2. Do different individual domains of prior knowledge correlate with recall?
3. Do these different domains of prior knowledge correlate with recall independently from other domains?

Second, the relationship between prior knowledge and recall of information supposedly is independent from other factors. So, to rule out additional factors influencing recall the next question is posed.

4. Do factors such as interest in a topic, common knowledge, and reading ability explain the correlation between domain-specific prior knowledge and recall of information?

Third, the ability to recall information ideally lasts longer than 1 week. Domain-specific prior knowledge should facilitate long-term recall.

5. Does domain-specific prior knowledge also facilitate long-term recall of information from a text about a complex topic?

## Materials and methods

### Ethic statement

This research was conducted in accordance with the ethical guidelines of the German Research Foundation (DFG) (Deutsche Forschungsgesellschaft, 1998). According to the guidelines of the ministry of education and cultural affairs in Lower Saxony, every study conducted at a public school has to be approved by the federal school board. This research was reviewed and approved by the federal school board of Lower Saxony, Germany. Due to the guidelines of the federal school board, the single schools had to give consent as well as the students' parents had to give written informed consent. Students volunteered and were free to omit single items or to drop out from the whole study any time they wanted. Students' anonymity was preserved, there was no individual-related feedback to the teachers or other participants. Students were informed about the specific procedures after the last session. All data was collected and analyzed anonymously.

### Participants

The 79 eleventh-grade students who participated in this study were attending different high schools in Lower Saxony, Germany and were surveyed during religion class. The group consisted of 43 girls and 36 boys, whose average age was 17 years and 1 month (*SD* = 0.62). At the long-term assessment date 12 weeks later only 51 students (30 girls and 21 boys) participated.

The topic of the survey was PND. Data collection took place during four school lessons on different days. During the first session personal data was gathered and students' prior knowledge was assessed. Afterwards, students were informed about PND in general with a short informational text (372 words) and their reading ability was assessed. During the second session students read a text with typical arguments for and against PND. This session often followed immediately after the previous session. The third session always took place 5–7 days after the second one. Students were asked about their interest in PND and to write down all the arguments they could remember from the text they read during the second session. Afterwards the students' common knowledge was assessed. Approximately 12 weeks after the third session a follow-up assessment was conducted. Students were asked again to write down all the arguments they could remember from the text they read during the second session.

### Prior knowledge

First, topic knowledge was assessed. Since it was expected that most students never had heard of PND, topic knowledge was gathered simply by asking students if they ever had heard of PND. While 76 students had not heard of it, three had. These three were excluded from further analysis.

The students' domain-specific knowledge was assessed by analyzing their responses on a questionnaire containing 39 items from the following domains relevant to PND: knowledge of the biological aspects of pregnancy, knowledge of medicine, knowledge of Christian values, knowledge of philosophical theories, knowledge of German federal law, knowledge of living conditions of people with special needs in Germany, and knowledge of consequences of abortion. For every domain there were at least five, and at most seven, items.

Each item was phrased as a statement (e.g., “A diagnosis is the assignment of a disease to an ordained constellation of symptoms.”) Students judged on a five-point Likert scale whether each statement was certainly right, maybe right, I don't know, maybe wrong or certainly wrong. If they correctly chose certainly right/certainly wrong, they scored two points; if they correctly chose maybe right/maybe wrong, they scored one point. If students chose I don't know, they got no points. If they incorrectly chose maybe right/maybe wrong or certainly right/certainly wrong they earned minus one and minus two points, respectively. For each domain, points were aggregated for a domain score. These domain scores were added together to obtain a total score of prior domain knowledge (hereafter referred to as *total domain knowledge*). Cronbachs α for the total domain knowledge score was α = 0.54. However, Schmitt (1996) emphasized that internal consistency should not be the only criteria for the usefulness of a measure. Further thoughts on this measure and its low reliability will follow in the Discussion.

### Text with arguments

The text the students read for recall had 1455 words and comprised seven arguments for and nine arguments against PND. Arguments were chosen from public discussions and dealt with short-term and long-term consequences of the use or non-use of PND for the parents, the unborn child, and society. To avoid effects by presenting pro- or contra-arguments first we counterbalanced whether a text started with pro- or contra-arguments. Students were instructed to read the text carefully.

### Recall

Students were asked to write down all the arguments they could remember that were presented in the text 1 week after reading the text and again 12 weeks later. Each time this took ~5 min. For each argument recalled correctly students received one point. Points were summed up for a total recall score. After 1 week students recalled between zero and six arguments with a mean of 2.5 (*SD* = 1.7). After 12 weeks—on the fourth assessment date—students recalled between zero and six arguments with a mean of 1.7 arguments (*SD* = 1.5). The indices for Inter-rater reliability (calculated on the basis of a part of the questionnaires) are *r* = 0.77 (*p* < 0.01; *n* = 35) for recall after 1 week and *r* = 0.77 (*p* < 0.01; *n* = 15) for recall after 12 weeks.

### Common knowledge

Common knowledge was assessed using the common knowledge part of the Intelligence Structure Test 2000 R (I-S-T 2000 R; Liepmann et al., 2007). This part of the test consists of 73 items but was shortened for this study to 33 randomly chosen items. Students were given 15 min to complete this test. For each correct answer they received one point. The points were summed up for a total common knowledge score (Cronbachs α = 0.55 for the scale used in this study, Liepmann et al. report α = 0.93 for the whole knowledge scale).

### Interest

As Renninger (2000) suggests, interest was assessed with three questions. Students rated on a five-point Likert scale their interest in PND, how relevant they considered this topic and how much they knew about it. The Likert scale ranged from *not at all* to *very much*. A mean was calculated for the three questions (Cronbachs α = 0.67).

### Reading ability

Reading ability was assessed with a reading speed and reading comprehension test (Schneider et al., 2007). The test provides two scores, one for reading speed and one for reading comprehension (retest reliability reported in the test manual: *r* = 0.84 and 0.87, respectively).

### Procedure

Data assessment lasted four school lessons. First students were greeted and informed about the purpose of the questionnaires. Their personal information was gathered, including prior topic knowledge. Then prior knowledge was assessed. To inform students on the topic a short informational text was given, after that their personal interest was assessed. In the second lesson, mostly following directly after the first, students read the text on arguments in favor and against PND. This took about 30 min. Their reading ability was tested as well. The third lesson was never earlier than at least 5 days after the second. Students were asked to recall all arguments they remembered from the text in lesson two. They had as much time as they needed. Then their common knowledge was assessed. The fourth lesson took place about 12 weeks after the third lesson. Again students were asked to recall all arguments from the text. Afterwards their questions regarding this study were answered.

## Results

### Correlations between prior knowledge and recall

First, the relationship between total domain knowledge and recall of a complex topic (i.e., PND) was assessed. The correlation between total domain knowledge and recall was significant (see Table 1). Domain-specific prior knowledge seemed to facilitate recall of complex topics. Second, the seven different domains of prior knowledge and their individual relationships to recall were examined. To decide when this research question is answered positively, it had to be determined how many correlations out of seven had to be significant. With the calculations to inflation of error type 1 as a decision making basis (see Appendix) it was decided, that at least two correlations had to be significant. The likelihood of at least two correlations being detected as significant if no relationship existed between the domains of prior knowledge and recall (error type 1) is *p* = 0.044, that is, it is smaller than α = 0.05. So it was determined that at least two correlations out of seven between the domains of prior knowledge and recall had to be significant to answer the second research question positively. For correlations between the seven domains of prior knowledge and recall see Table 1. The correlations between three domains (knowledge of medicine, knowledge of Christian values and knowledge of German federal law) and recall became significant (of seven correlations) and supported the hypothesis that there would be a significant impact of individual domains of prior knowledge on recall. To ensure that the different domains correlated independently with recall—as was the third research question—, each domain was correlated with recall while examining the other six domains. Of these seven partial correlations, two (knowledge of medicine and knowledge of German federal law) were significant (see Table 1). Third, the different domains of prior knowledge correlated independently with recall.

**Table 1:** **Correlations between prior knowledge and recall at third and fourth sessions**.

	**Recall after 1 week Controlling…**						**Recall after 12 weeks Controlling…**					
		**Other domains**	**Interest**	**Common knowledge**	**Reading speed**	**Reading comprehension**		**Other domains**	**Interest**	**Common knowledge**	**Reading speed**	**Reading comprehension**
Total prior knowledge	0.350^**^	−	0.317^**^	0.357^**^	0.344^**^	0.296^**^	0.316^**^	−	0.298^*^	0.298^*^	0.314^*^	0.287^*^
Knowledge of biology	0.039	−0.115	0.009	0.012	0.015	0.020	0.025	−0.029	0.054	0.003	0.014	0.000
Knowledge of medicine	0.221^*^	0.087	0.232^*^	0.240^*^	0.228^*^	0.195	0.251^*^	0.140	0.211	0.184	0.220	0.196
Knowledge of Christian values	0.194^*^	0.317^*^	0.152	0.169	0.163	0.122	0.213	0.206	0.225	0.238	0.239	0.229
Knowledge of philosophical concepts	0.063	0.078	0.119	0.064	0.062	0.051	0.017	−0.078	0.101	−0.002	0.022	−0.016
Knowledge of German federal law	0.394^**^	0.376^**^	0.365^**^	0.403^**^	0.401^**^	0.365^**^	0.221	0.199	0.197	0.222	0.219	0.210
Knowledge of special needs	0.068	−0.212	0.075	0.117	0.099	0.064	0.114	−0.028	0.080	0.095	0.125	0.106
Knowledge of consequences of abortion	0.166	0.110	0.102	0.176	0.157	0.127	0.215	0.155	0.116	0.195	0.181	0.179

* *α = 0.05*,

** *α = 0.01*.

### Alternative factors possibly explaining the correlation between prior knowledge and recall

Partial correlations between prior knowledge and recall were explored while examining the impact of interest, common knowledge, reading speed and reading comprehension. The correlation between total domain knowledge and recall with interest as a control item was significant (*r* = 0.317). Partial correlations between the seven prior knowledge domains and recall with interest as a control item were examined as well. Of these seven partial correlations two (knowledge of medicine and knowledge of German federal law) were significant (see Table 1). So, even with interest as a control item the correlations between some domains of prior knowledge and recall remained significant. To get further insights, a regression analysis with interest as a moderating variable, recall as the outcome variable and total prior knowledge as the independent variable was conducted. Total prior knowledge predicts recall. There is no significant interaction effect with interest (see Table 2).

**Table 2:** **Linear model of predictors [CI] of recall**.

	***b***	***SE B***	***t***	***P***
Constant	2.51 [2.10, 2.91]	0.205	12.28	0.00
Total prior knowledge (centered)	0.06 [0.02, 0.12]	0.025	2.60	0.01
Interest (centered)	0.67 [0.15, 1.20]	0.264	2.57	0.01
Total prior knowledge × interest	0.03 [−0.02, 0.09]	0.028	1.33	0.19

*R^2^ = 0.19*.

Next, common knowledge was taken as a control item in partial correlations between prior knowledge and recall. The correlation between total domain knowledge and recall was significant (*r* = 0.357). Again, there was a significant partial correlation between knowledge of medicine as well as knowledge of German federal law and recall (see Table 1). These outcomes lead to the conclusion that common knowledge does not explain correlations between prior knowledge and recall.

Finally, reading ability was analyzed as an alternative factor explaining correlations between prior knowledge and recall. Reading speed as a control item in partial correlations between total domain knowledge and recall did not decrease the correlation (*r* = 0.344). As before, there was a significant partial correlation between knowledge of medicine as well as knowledge of German federal law and recall (see Table 1). This implies that reading speed did not explain correlations between prior knowledge and recall. For reading comprehension as a control item in partial correlations between prior knowledge and recall there was a different outcome. Reading comprehension did not explain the correlation between total domain knowledge and recall (*r* = 0.296). However, as a control item in partial correlations between the seven prior knowledge domains and recall only one domain (knowledge of German federal law) correlated significantly with recall. Regarding accumulated error type 1 this occurrence has the likelihood of *p* = 0.302. Reading comprehension might explain correlations between the seven domains of prior knowledge and recall.

### Correlations between prior knowledge and long-term recall

The relationship between prior knowledge and long-term recall after 12 weeks during the fourth session was analyzed. Because these analyses involved only 51 subjects, the validity of the findings is slightly reduced. While assessing recall during the third session, correlations between long-term recall as well as total domain knowledge and the seven different domains of prior knowledge was assessed. The correlation between total domain knowledge and recall during the fourth session was *r* = 0.316 and significant (see Table 1). Of the seven prior knowledge domains only the correlation between medical knowledge and recall was significant. Therefore, the assumption that the different domains of prior knowledge correlate separately and individually with long-term recall has to be rejected. This was supported by the next finding: Partial correlations between the particular prior knowledge domains and long-term recall with all the other domains of prior knowledge as control items did not produce any significant correlation (see Table 1).

Interest, common knowledge and reading ability did not explain the relationship between recall during the fourth session and prior knowledge. Partial correlations between long-term recall and total domain knowledge with interest, common knowledge, reading speed and reading comprehension as control items were found. None of these explained the correlations between total domain knowledge and recall during the fourth session (see Table 1). The partial correlations between the seven different prior knowledge domains and recall with these four factors as control items were found as well (see Table 1). There was no significant partial correlation to be found.

## Discussion

The central question in this study was whether recall of an ill-structured topic from social sciences is supported by prior knowledge. Most research on prior knowledge has been conducted on science or mathematical topics. Topics from social sciences often cannot be matched to one single domain of prior knowledge. Instead, different domains might be related to one topic. In this study the bioethical topic chosen was PND. Prior knowledge of different domains was assessed along with interest in the topic, common knowledge and reading ability. Students read a text on PND presenting arguments in favor of it and against it. One week later and again after 12 weeks, their recall of arguments was assessed. The results showed that the students' total domain knowledge correlated with their recall ability after 1 week. Other factors possibly facilitating recall after 1 week such as interest, common knowledge, and reading ability did not explain this correlation. Because topics in social sciences sometimes cannot be allocated exclusively to one domain of prior knowledge, different domains of prior knowledge were investigated to determine whether they facilitated recall individually. The results showed that three of seven domains correlated with recall. Since these domains were all related to one topic, they might have correlated with each other as well, thus explaining correlations with recall. The results showed that while examining all the domains, two of seven still correlated with recall and therefore were independent from the other domains. Different domains of prior knowledge correlated at the same time with recall of one topic. Because this broad domain-specific prior knowledge might be similar to common knowledge, common knowledge might explain the relationship between the different prior knowledge domains and recall. However, the results showed that common knowledge is different from prior knowledge and does not explain its relation to recall. The same pattern occurred with reading ability. Interest, as well, does not explain the relation between prior knowledge and recall. Though both predict recall, interest does not interact with prior knowledge. With PND as a topic, different domains of prior knowledge seemed to be separately related to recall. Common knowledge did not explain these partial correlations although domain-specific prior knowledge is broader because more domains are involved simultaneously. This is considered a central finding of this study, since prior knowledge is conceptualized so differently and sometimes broadly (cf. Dochy, 1992; Alexander et al., 1994b; Tobias, 1994; Dochy et al., 1999). Although interest is known to be relevant for recall as is prior knowledge, it did not explain correlations between the different domains of prior knowledge and recall. In addition, it does not interact with prior knowledge. Both are factors predicting recall independently. So even with a complex topic like PND, prior knowledge and interest are both important.

An important limitation to the findings of this study are the low reliability scores for the measurement of prior knowledge (Cronbachs α = 0.54) and of common knowledge (Cronbachs α = 0.55). For different reasons it was decided that the results in this study could be carefully interpreted anyway. One reason was the found results which were consistent with the expected results. Low correlations with other variables should be interpreted most carefully, but significant correlations can be considered as argument for given reliability and validity. In addition, Schmitt (1996) argued that even if the alpha level is low, one can still interpret results from this measure, if it is done with caution. A third reason is that questionnaires collecting knowledge often have lower alpha levels (cf. Voss et al., 2011; Wilhelm et al., 2014).

Some questions arise from these findings. Since only three of the seven domains of prior knowledge correlated with recall of arguments about PND, one could ask why the other domains did not correlate as well. This might have been due to the construction of the questionnaire for prior knowledge, which could be improved in later studies. This assumption is supported by the finding that reading comprehension interfered in the correlation between medical knowledge and recall. As Dochy et al. (1999) point out, the way prior knowledge is assessed affects whether a relationship between prior knowledge and recall is found. In this study domain-specific prior knowledge was assessed in a way similar to that in other studies (cf. Alexander et al., 1994a, 1995; DeMarie et al., 2004; Levin and Arnold, 2004); since the method is appropriate there, non-significant correlations between some domains of prior knowledge and recall in this study might have other reasons which need to be considered. Perhaps the domains of prior knowledge differ in their distance to the topic. Some might be more closely related than others to the topic of PND. Maybe this relatedness is mediated by students' perception of the topic and its relevant domains. Only one group of students was surveyed; a different group might produce slightly different results. Further research with a different group of students might be worthwhile.

Another question arising from these findings is whether they can be generalized to other topics. Since these findings come from learning about the rather rare topic of PND, other topics from the social sciences and the prior knowledge relevant to them should be investigated. As most topics stemming from social sciences are complex in their structure, deciding on the domains in which prior knowledge might be helpful is challenging. However, a topic like PND is compatible with many school subjects due to the different domains to which it is related. In Germany, PND is discussed in religion as well as in biology classes, which allows teaching these subjects to be interdisciplinary. Research on interdisciplinary teaching is rare.

The second important finding of this study is that recall after a longer period of time (here 12 weeks) still is related to prior knowledge. In other studies a follow-up test after only 1 week was administered (cf. Hall and Edmondson, 1992; Gilabert et al., 2005). However, at school a topic covered in class might become relevant to learning another topic weeks or months later. Students need to be able to recall knowledge after a much longer period of time than 1 week. The findings in this study show that students with more domain-specific prior knowledge have an advantage over students with less knowledge. Different from the findings described above, only domain-specific prior knowledge in general facilitated recall. The seven domains of prior knowledge did not correlate with later recall individually. The reasons for these findings might be methodical and/or related to the long period between learning and recall. A methodical reason might be the relatively small number of only 51 participants at the last assessment session. Correlations are small and therefore might not be significant with such a small number of participants. Another explanation is that after 12 weeks the particular memory students had of the text because of their domain-specific prior knowledge had faded and only the total domain knowledge facilitated recall. To rule out the first explanation another study with more participants would be helpful. However, interest, common knowledge and reading ability did not explain the relationship between total prior knowledge and later recall. This supports the hypotheses that prior knowledge is crucial to recalling newly learned information even after a period of 12 weeks.

If further research is conducted, some possible improvements should be considered. In this study, prior knowledge was not explicitly activated before learning. The importance of prior knowledge activation is well-known (e.g., Alvermann et al., 1985; Schmidt et al., 1989). In this study, prior knowledge activation was not emphasized as it was unclear which prior knowledge would be vital. Another interesting aspect is students' feelings toward the topic. Interest does not cover emotions like distress, disgust or delight. Research on attitudes in educational contexts shows that, students learn information regardless of their own attitude (Henk and Holmes, 1988; Hollingsworth and Reutzel, 1990). However, research on emotions during the learning process shows that emotions do influence learning (e.g., Ainley et al., 2005; Holstermann et al., 2012). With a bioethical topic, emotions might have some influence on the learning process as well.

In the end, the question arises whether scientific and social topics really are different from each other considering prerequisites and the learning process as well. Maybe research on prior knowledge in scientific topics focusing only on one domain did not consider that even in scientific topics more than one domain might be relevant. e.g., Learning on black holes and Stephen Hawking might not only require prior knowledge in the domain of physics but also some knowledge on which Stephen Hawking is. On the other hand, learning and solving problems through discussions seems to require more heuristic strategies and less algorithmic strategies—which might be different with learning in and problems from scientific contexts. So, in this regard, social and scientific topics might be different. This research at least shows that multiple domains are helpful in understanding a social topic.

### Conflict of interest statement

The authors declare that the research was conducted in the absence of any commercial or financial relationships that could be construed as a potential conflict of interest.

## Appendix

The likelihood of α-error or type I error (detecting a significant correlation that is not present) increases with the number of correlations executed. The possibility that at least one correlation of seven is—by error—significant is *p*_(1)_ = 0.257.

The binominal formula

$$p\;\left(k\right)=\sum_{k\;=\;0}^{n}\left(\begin{array}{c} n\\ k \end{array}\right)x^{k}a^{n-k}=\;\frac{n!}{k!\left(n-k\right)!}\;\times p^{k}\;\times {\left(1-p\right)}^{n-k};$$

where *n* = 7, *n*! = 7 × 6 × 5 × 4 × 3 × 2 × 1, *p* = 0.05,

*k* = number of correlations incorrectly detected as significant, was used to calculate the likelihood of having one, two, three…correlations becoming incorrectly significant.

The likelihood of incorrectly detecting correlations as significant as well as the accumulated likelihood of the events is shown in Table A1. The accumulated likelihood was used as a basis for decision-making. The likelihood of the event of incorrectly deciding that a number of *k* correlations was significant had to be ≤0.05.

**Table A1:** **Likelihood of incorrectly detecting correlations**.

***p*(*k*)**	**Likelihood of incorrectly detecting *k* correlations**	**Likelihood of incorrectly detecting at least *k* correlations**
*p* (1)	0.257282	0.301659
*p* (2)	0.040623	0.044380
*p* (3)	0.003563	0.003757
*p* (4)	0.000188	0.000194
*p* (5)	0.000006	0.000006
*p* (6)	0.000000	0.000000
*p* (7)	0.000000	0.000000
